# Beef tenderness and intramuscular fat proteomic biomarkers: muscle type effect

**DOI:** 10.7717/peerj.4891

**Published:** 2018-06-07

**Authors:** Brigitte Picard, Mohammed Gagaoua, Marwa Al-Jammas, Leanne De Koning, Albéric Valais, Muriel Bonnet

**Affiliations:** 1 Université Clermont Auvergne, INRA, VetAgro Sup, UMR Herbivores, Saint-Genès-Champanelle, France; 2 Institut Curie Centre de Recherche, Université de recherche PSL, Plateforme RPPA, Paris, France; 3 S.I.C.A. Rouge des Prés, Domaines des rues, Chenillé-Champteussé, France

**Keywords:** Cattle, Muscle type, Biomarkers, RPPA, Proteomics, Biological mechanisms

## Abstract

Tenderness and intramuscular fat content are key attributes for beef sensory qualities. Recently some proteomic analysis revealed several proteins which are considered as good biomarkers of these quality traits. This study focuses on the analysis of 20 of these proteins representative of several biological functions: muscle structure and ultrastructure, muscle energetic metabolism, cellular stress and apoptosis. The relative abundance of the proteins was measured by Reverse Phase Protein Array (RPPA) in five muscles known to have different tenderness and intramuscular lipid contents: *Longissimus thoracis* (LT), *Semimembranosus* (SM), *Rectus abdominis* (RA), *Triceps brachii* (TB) and *Semitendinosus* (ST). The main results showed a muscle type effect on 16 among the 20 analyzed proteins. They revealed differences in protein abundance depending on the contractile and metabolic properties of the muscles. The RA muscle was the most different by 11 proteins differentially abundant comparatively to the four other muscles. Among these 11 proteins, six were less abundant namely enolase 3 (ENO3), phosphoglucomutase 1 (PGK1), aldolase (ALDOA), myosin heavy chain IIX (MyHC-IIX), fast myosin light chain 1 (MLC1F), triosephosphate isomerase 1 (TPI1) and five more abundant: Heat shock protein (HSP27, HSP70-1A1, αB-crystallin (CRYAB), troponin T slow (TNNT1), and aldolase dehydrogenase 1 (ALDH1A1). Four proteins: HSP40, four and a half LIM domains protein 1 (FHL1), glycogen phosphorylase B (PYGB) and malate dehydrogenase (MDH1) showed the same abundance whatever the muscle. The correlations observed between the 20 proteins in all the five muscles were used to construct a correlation network. The proteins the most connected with the others were in the following order MyHC-IIX, CRYAB, TPI1, PGK1, ALDH1A1, HSP27 and TNNT1. This knowledge is important for understanding the biological functions related to beef tenderness and intramuscular fat content.

## Introduction

Tenderness is the most important attribute of beef eating sensory quality affecting consumer’s acceptability (Miller et al., 2001). The wide inconsistency of this quality is a limiting factor for consumer product’s acceptability and is the main reason for consumer’s dissatisfaction and reduction in beef consumption. For years, several studies have shown that most consumers are willing to pay more for steaks that are “guaranteed tender” (Boleman et al., 1997; Miller et al., 2001). Inconsistency in beef tenderness has also been identified as a major problem for the beef industry (Brooks et al., 2000; Lorenzen et al., 1999; Picard et al., 2014). In addition, the degree of marbling defined as the amount and distribution of intramuscular fat (IMF) in the *longissimus lumborum* muscle, is the primary factor used to determine beef quality grade. For example, in United States, Japan or Korea, the challenge to the beef cattle industry is the production of meat with high marbling score, which is the most economically important and valuable trait. In European countries, an IMF of 3–4% in beef is required for a good sensory appreciation by consumers (Bonnet et al., 2010; Hocquette et al., 2010). Moreover, IMF also influences meat flavor, lipid oxidation, contributes to beef color and affects the juiciness and tenderness of meat (Bonnet et al., 2010; Gagaoua, Picard & Monteils, 2018c; Hocquette et al., 2010).

Tenderness and IMF appear to represent major quality traits to be controlled in beef. Currently, these criteria are evaluated only after slaughter, by sensory analysis panel and/or mechanical measurements for tenderness, and by chemical analyzes for IMF. These methods are time consuming and costly, so there is a need to develop new efficient and non-invasive tools. Thus, for several years, numerous functional genomic studies have been developed to identify biomarkers of beef qualities, mainly tenderness (for review: Picard & Gagaoua, 2017; Picard, Gagaoua & Hollung, 2017b; Picard et al., 2015) and more recently of meat fat deposition (for review: Ceciliani et al., 2018). Among these methods, proteomics appeared to be a relevant technique to analyze protein changes during *post-mortem* ageing and to reveal proteins biomarkers of tenderness (for review: Ouali et al., 2013; Picard & Gagaoua, 2017; Picard, Gagaoua & Hollung, 2017b; Picard et al., 2015) or fat deposition (for review: Ceciliani et al., 2018). The devolvement of comparative proteomic analyses allowed the identification of a list of proteins considered as potential biomarkers of beef tenderness or fat deposition. The list of proteins was further used to understand the biological basis underpinning the conversion of muscle into meat (Gagaoua et al., 2018a; Gagaoua et al., 2015b; Gagaoua et al., 2015c). However, most of the studies have been performed on the *Longissimus thoracis* muscle (LT, mixed fast oxido-glycolytic), which is a valuable cut for steak. Few studies looked at the *Semitendinosus* muscle (ST, fast glycolytic) or other muscles (Picard & Gagaoua, 2017). The first proteomic investigations compared LT and ST muscles (Gagaoua et al., 2015b; Guillemin et al., 2011a; Picard et al., 2014). The bioinformatic analysis showed that the network of beef tenderness was different between LT and ST muscles (Guillemin et al., 2011a). Some proteins such as µ-calpain (involved in proteolysis), αB-crystallin (Hsp), Peroxiredoxin 6 (Prdx6) and Park7 known also as DJ-1(involved in oxidative stress resistance), Myosin light chains (MYL1), Myosin heavy chain IIA (MYH2) and Myosin binding protein H (MYBP-H) (involved in structure and contraction), were related with tenderness in the same way in the two muscles. However, some relationships were muscle specific (Picard et al., 2014). Later on, comparisons between LT and *Rectus abdominis* were reported (Gagaoua et al., 2017a). Among the quantified biomarkers, nearly most proteins (18 from 23) belonging to six different biological functions: heat shock proteins; muscle fiber; energy metabolism; proteolysis; oxidative resistance and apoptosis were different between the two muscles. Very recently, both proteomic and peptidomic approaches on New Zealand-raised Angus steers using four muscles (*Semitendinosus*, *Longissimus thoracis* et *lumborum*, *Psoas major* and *Infraspinatus*) revealed further knowledge (Clerens et al., 2016). Protein profiling based on two-dimensional electrophoresis showed that the overall profiles were similar, but among muscle types, significant intensity differences were observed in 24 protein spots.

Thus, the aim of the present study was to analyze for the first time the effect of muscle type on the relative abundance of 20 proteins considered as biomarkers of tenderness and/or IMF content in five muscles: *Longissimus thoracis* (LT), *Semimembranosus* (SM), *Rectus abdominis* (RA), *Triceps brachii* (TB) and *Semitendinosus* (ST). The proteins were quantified on 101 Protected Designation Origin (PDO) Maine-Anjou cattle (Gagaoua et al., 2017a; Gagaoua et al., 2017b) by the means of Reverse Phase Protein Array (RPPA) (Gagaoua et al., 2018a). We further built correlation networks among the 20 proteins for all the muscles to reveal robust correlations, *i.e.* found for the different muscles using standardized data. The results produced for the first time new insights that could be applied for a better understanding of the biological pathways involved in meat quality according to the type of muscle.

## Materials and Methods

### Animals, handling and slaughtering

A total of 101 cattle from the French official sign of quality PDO (Protected Designation Origin) Maine-Anjou, using “Rouge des Prés” breed, were collected (Gagaoua et al., 2018b). This breed is the result of a crossbreeding initiated 100 years ago between the Durham English bulls and the local Mancelle breed. From the Mancelle, the breed holds its rusticity, its vigor and its predisposition to fattening. The Durham brought its precocity. The PDO Maine-Anjou beef is deemed by its marble, sustained red color, tenderness and intensity of flavors. This official quality mark is involved in a research of predictors of tenderness and fatness of the meat during these recent years (Gagaoua et al., 2017a; Gagaoua et al., 2017b).

Before slaughter, all animals were food deprived for 24 h and had free access to water. All the animals were slaughtered in the same industrial abattoir (Charal, Sablé sur Sarthes, France), stunned using captive-bolt pistol prior to exsanguination and dressed according to standard commercial practices. Slaughtering was performed in compliance with the French welfare regulations and respecting EU regulations (Council Regulation (EC) No. 1099/2009). After slaughter, the carcasses were graded according to the European beef grading system (CE 1249/2008). The carcasses were not electrically stimulated and they were chilled between 2 to 4 °C until 24 h *post-mortem*.

### Muscle sampling

Five muscles: *Longissimus thoracis* (LT), *Semimembranosus* (SM), *Rectus abdominis* (RA), *Triceps brachii* (TB) and *Semitendinosus* (ST), were excised from the right-hand side of the carcass of each animal 1 h after slaughter. For proteins analysis, muscle samples were frozen in liquid nitrogen and stored at –80 °C until analysis.

### Measure of the relative abundance of proteins

The relative abundances of 20 proteins biomarkers of tenderness and/or intramuscular fat (Picard et al., 2017a) were measured on the five muscles by the Reverse Phase Protein Array (RPPA) using specific antibodies as recently described (Gagaoua et al., 2018a). The specificity of the 20 antibodies on bovine muscle (Table 1) and their conditions of use have been checked using western blotting (Gagaoua, Terlouw & Picard, 2017c).

**Table 1:** List of the 20 proteins quantified using the Reverse Phase Protein Array (RPPA) technique. The suppliers and conditions for each primary antibody used in this study after western blotting validation are given.

Protein biomarkers name (*gene*)	Uniprot ID	Monoclonal (Mo) or Polyclonal (Po) antibodies references	Antibody dilutions
***Metabolic enzymes***			
Malate dehydrogenase (*MDH1*)	**P40925**	Mo. anti-pig Rockland 100 − 601 − 145	1/1000
β-enolase 3 (*ENO3*)	**P13929**	Mo. anti-human Abnova Eno3 (M01), clone 5D1	1/30 000
Retinal dehydrogenase 1 (*ALDH1A1*)	**P48644**	Po. anti-bovine Abcam ab23375	1/500
Triosephosphate isomerase (*TPI1*)	**Q5E956**	Po. anti-human Novus NBP1-31470	1/50 000
Phosphoglycerate kinase 1 (*PGK1*)	**Q3T0P6**	Po. anti-human Abcam ab90787	1/5000
Fructose-bisphosphate aldolase (*ALDOA*)	**A6QLL8**	Po. anti-human Sigma AV48130	1/4000
Glycogen phosphorylase (*PYGB*)	**Q3B7M9**	Po. anti-human Santa Cruz SC-46347	1/250
***Heat shock proteins***			
αB-crystallin (*CRYAB*)	**P02511**	Mo. anti-bovine Assay Designs SPA-222	1/1000
Hsp20 (*HSPB6*)	**O14558**	Mo. anti-human Santa Cruz HSP20-11:SC51955	1/500
Hsp27 (*HSPB1*)	**P04792**	Mo. anti-human Santa Cruz HSP27 (F-4):SC13132	1/3000
Hsp40 (*DNAJA1*)	**P31689**	Mo. anti-human Santa Cruz HSP40-4 (SPM251):SC-56400	1/250
Hsp70-1A (*HSPA1A*)	**Q27975**	Mo. anti-human RD Systems MAB1663	1/1000
***Oxidative proteins***			
Peroxiredoxin6 (*PRDX6*)	**P30041**	Mo. anti-human Abnova PRDX6 (M01), clone 3A10-2A11	1/500
***Structural proteins***			
MLC-1F (*MYL1*)	**P05976**	Po. anti-human Abnova MYL1 (A01)	1/1000
Myosin heavy chain-IIx (*MyHC-IIx*)	**P12882**	Mo anti-bovine Biocytex 8F4	1/500
Troponin T, slow skeletal muscle (*TNNT1*)	**Q8MKH6**	Po. anti-human Sigma SAB2102501	1/4000
Titin (*TTN*)	**Q8WZ42**	Mo. anti-human Novocastra NCL-TITIN	1/100
Tubulin alpha-4A chain (*TUBA4A*)	**P81948**	Mo anti-human Sigma T6074	1/1000
***Cell death and protein binding***			
Tripartite motif protein 72 (*Trim72*)	**E1BE77**	Po. anti-human Sigma SAB2102571	1/2000
Four and a half LIM domains 1 (*FHL1*)	**Q3T173**	Po. anti-human Sigma AV34378	1/5000

The relative abundances of proteins were determined according to the following procedure. First, raw data were normalized using NormaCurve (Troncale et al., 2012), a SuperCurve-based method that simultaneously quantifies and normalizes reverse phase protein array data for fluorescent background per spot, a total protein stain and potential spatial bias on the slide. Next, each RPPA slide was median centered and scaled (divided by median absolute deviation). We then corrected for remaining sample loadings effects individually for each array by correcting the dependency of the data for individual arrays on the median value of each sample over all the arrays using a linear regression.

### Statistical analyses

Statistical analyses were performed using SAS statistical software (SAS 9.1, SAS Institute INC, Cary, NC, USA) and /or XLSTAT 2017.19.4 (AddinSoft, Paris, France). Before analysis, raw data means were scrutinized for data entry errors and outliers. The PROC GLM procedure of SAS was used to study the muscle effect on the relative abundances of the proteins. Significant differences among muscles were performed using Tukey’s test at a significance level of *P* < 0.05. Subsequently a heat-map was produced using the same normalized data.

The PROC CORR of SAS after *Z*-scores calculation was used to compute the Pearson’s correlations of coefficients among the 20 proteins. Then they allowed according to the procedure recently described by our group to construct a biological correlation network (Gagaoua et al., 2015b). Correlation values were included in the network if they were significant at *P* < 0.05 within each muscle alone and in the five muscles as one dataset.

A principal component analysis (PCA) was performed for the simultaneous visualization of the differences among the muscles and to represent the main proteins differing after an unsupervised clustering Heatmap analysis.

## Results

### Muscle type effect on the relative abundances of the 20 proteins

The results of variance analysis (Table 2) showed a highly significant effect of the type of muscle on the relative abundances of the studied proteins. Only 4 proteins, namely HSP40 (Heat shock protein), FHL1 (Four and a half LIM domains protein 1), PYGB (Glycogen phosphorylase B) and MDH1 (Malate dehydrogenase), did not differ between the 5 muscles.

**Table 2:** Comparison of the relative mean protein abundances between the five muscles.

Protein biomarkers name (*gene*)	Muscles						SEM		*P*-value^1^
	TB	ST	RA	SM	LT				
***Metabolic enzymes***									
Malate dehydrogenase (*MDH1*)	0.09	0.04	0.01	−0.11	0.07		0.02		ns
β-enolase 3 (*ENO3*)	0.22^bc^	0.58^a^	−1.22^d^	0.33^b^	0.10^c^		0.04		^***^
Retinal dehydrogenase 1 (*ALDH1A1*)	−0.16^bc^	−0.07^b^	0.73^a^	−0.28^c^	−0.15^bc^		0.03		^***^
Triosephosphate isomerase (*TPI1*)	0.04^c^	0.55^a^	−1.02^d^	0.31^b^	−0.03^c^		0.04		^***^
Phosphoglycerate kinase 1 (*PGK1*)	0.11^b^	0.39^a^	−0.95^c^	0.35^a^	0.11^b^		0.04		^***^
Fructose-bisphosphate aldolase (*ALDOA*)	−0.04^b^	0.26^a^	−0.24^c^	0.16^a^	−0.02^b^		0.02		^***^
Glycogen phosphorylase (*PYGB*)	0.08	0.11	−0.02	0.05	0.01		0.02		ns
***Heat shock proteins***									
αB-crystallin (*CRYAB*)	−0.15^bc^	−0.62^d^	1.03^a^	−0.21^c^	−0.02^b^		0.04		^***^
Hsp20 (*HSPB6*)	−0.23^c^	−0.25^c^	0.29^a^	0.01^b^	0.17^a^		0.03		^***^
Hsp27 (*HSPB1*)	−0.06^b^	−0.08^b^	0.61^a^	−0.44^c^	−0.04^b^		0.03		^***^
Hsp40 (*DNAJA1*)	−0.11	0.02	−0.05	0.06	−0.11		0.02		ns
Hsp70-1A (*HSPA1A*)	−0.20^c^	−0.36^c^	0.28^a^	0.17^ab^	0.08^b^		0.03		^***^
***Oxidative proteins***									
Peroxiredoxin6 (*PRDX6*)	0.16^a^	0.12^ab^	−0.03^b^	0.26^a^	−0.33^c^		0.03		^***^
***Structural proteins***									
MLC-1F (*MYL1*)	0.26^ab^	0.39^a^	−0.56^c^	0.08^b^	0.09^b^		0.03		^***^
Myosin heavy chain-IIx (*MyHC-IIx*)	0.27^b^	0.75^a^	−0.91^d^	0.06^b^	−0.21^c^		0.05		^***^
Troponin T, slow skeletal muscle (*TNNT1*)	0.09^b^	−0.97^d^	0.88^a^	−0.13^c^	0.08^b^		0.03		^***^
Titin (*TTN*)	0.30^a^	−0.33^c^	−0.05^b^	−0.31^c^	0.34^a^		0.03		^***^
Tubulin alpha-4A chain (*TUBA4A*)	0.05^a^	−0.03^ab^	0.10^a^	−0.02^ab^	−0.13^b^		0.02		^*^
***Cell death and signaling***									
Tripartite motif protein 72 (*Trim72*)	0.41^a^	−0.08^b^	0.01^b^	−0.11^b^	0.32^a^		0.02		^**^
Four and a half LIM domains 1 (*FHL1*)	0.12	−0.16	0.04	−0.03	0.01		0.03		ns

**Notes.**

a,b,c,d Least-square means in the same row with different superscript letters are significantly different (*P* < 0.05).

1 Significances.

ns not significant

* *P* < 0.05.

** *P* < 0.01.

*** *P* < 0.001.

Muscle abbreviations: TB *Triceps brachii* ST *Semitendinosus* RA *Rectus abdominis* SM *Semimembranosus* LT *Longissimus thoracis*

**Figure 1:**
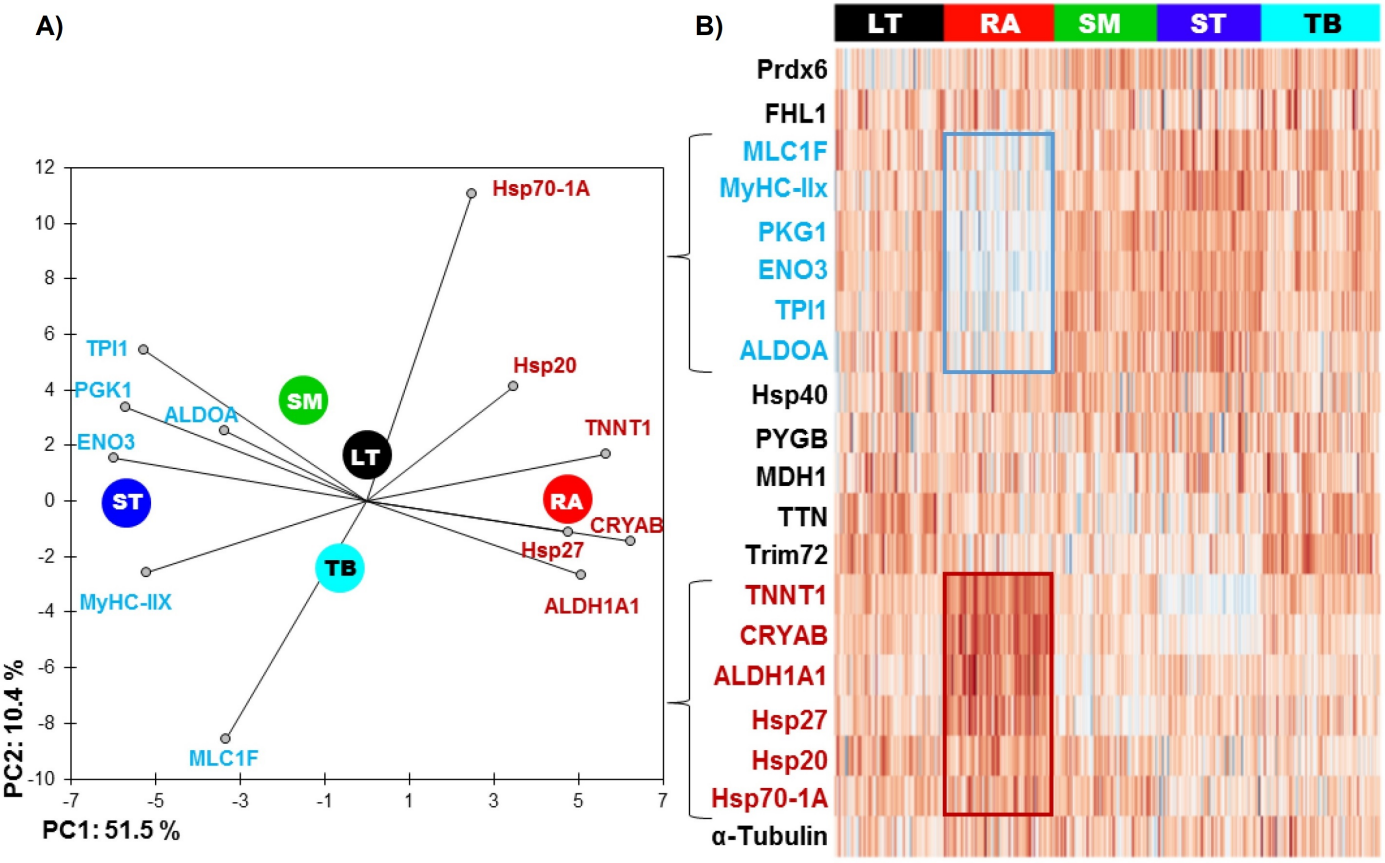
(A) Principal component analysis (PCA) of the most discriminating proteins between the muscles using the RA muscle as a reference and (B) unsupervised hierarchical clustering heatmap highlighting the proteins, which are low (blue) and high (red) abundant. The graph is showing the differences in the relative abundances of the proteins studied namely between RA and ST muscle. Colors correspond to the log2 transformed values of protein fold-change.

The RA muscle was the most different from the others (Table 2 and Fig. 1), with 11 out of 20 proteins whose abundance was significantly different compared to other muscles. Six proteins had significantly lower abundance values: ENO3 (Enolase 3), PGK1 (Phosphoglucomutase 1), TPI1 (Triosephosphate isomerase 1), ALDOA (Aldolase), MyHC-IIX (Myosin heavy chain IIX) and MLC1F (Fast myosin light chain 1), and 5 proteins were more abundant: ALDH1A1 (Aldolase dehydrogenase 1), HSP70-1A1, HSP27, CRYAB (αB-crystallin) and TNNT1 (Troponin T slow).

The ST muscle was characterized by high abundances of ENO3, TPI1, ALDOA, MyHC-IIX and low abundances of TTN (Titin), Hsp20, Hsp701A1 and CRYAB (Table 2 and Fig. 1).

**Figure 2:**
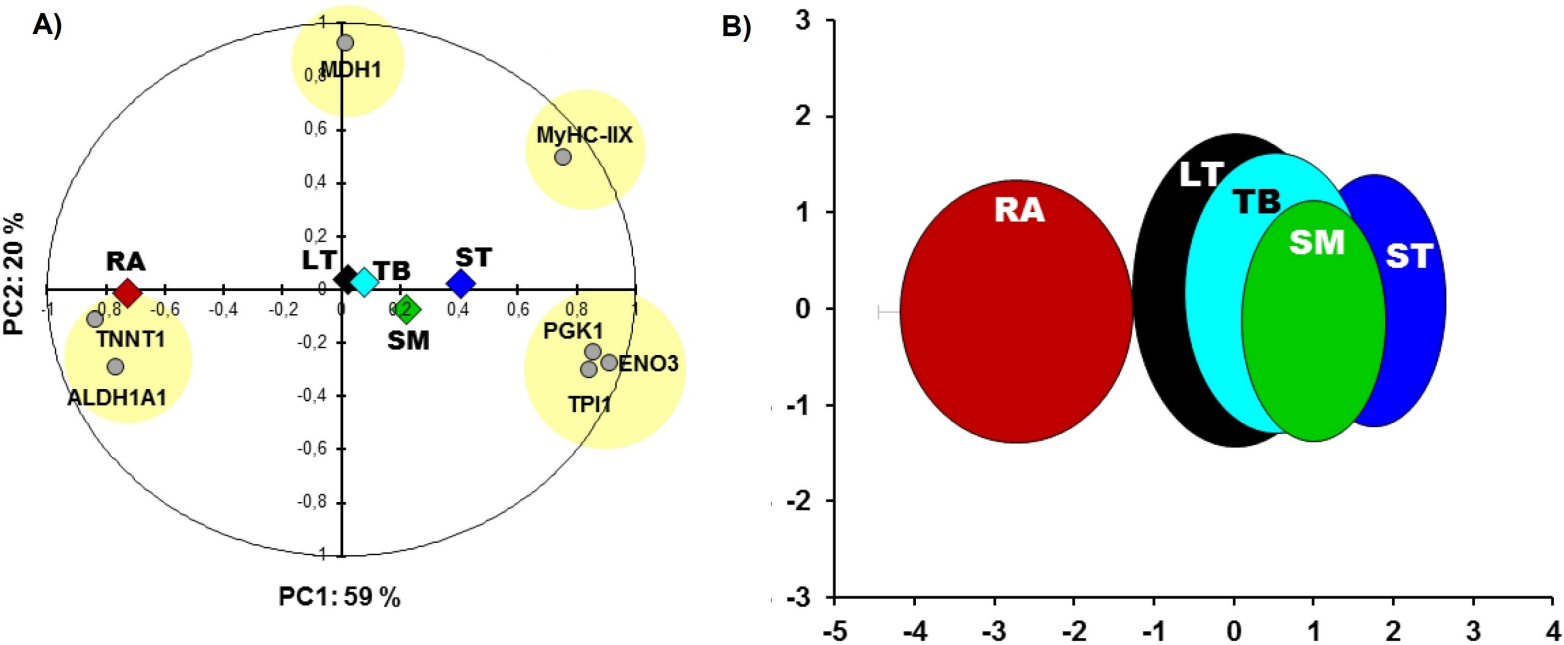
Principal component analysis (PCA). (A) Comparisons between the five muscles according to proteins of metabolic (MDH1, ALDH1A1, PGK1, ENO3, TPI1) and contractile properties (MyHC-IIX and TNNT1). The projections of the mean individuals for each muscle are shown in the same PCA by their barycenter’s and corresponding color on both axes. (B) the bi-plot of the projection of the individuals of the five muscles: RA (red), LT (black), TB (cyan), SM (green) and ST (blue) that are encircled in ellipses (x,y-means ± x,y-standard deviation (SD) as in Gagaoua, Picard & Monteils (2018c)) using the corresponding schematic colors. The overall Kaiser-Mayer-Olkin score of the PCA was 0.73 (Bartlett’s test of sphericity was significant, *P* < 0.001). The eigenvalues of PC1 and PC2 are 4.2 and 1.4, respectively.

RA and ST muscles were in this study opposite on protein abundances as can be easily seen on the Fig. 1. Proteins related to the contractile and metabolic properties (MDH) and quantified in this study (MyHC-IIX, PGK1, ENO3, TPI1, TNNT1 and ALDH1A1) allowed a clear discrimination of the muscles by the means of principal component analysis, specifically when RA and ST muscles were compared (Fig. 2).

The results of Table 2 further showed that the abundances of several proteins such as TTN, ENO3, HSP27, PGK1, ALDOA, CRYAB, TRIM72, TNNT1, TPI1, ALDH1A1, and MLC-1F were not different between LT and TB muscles. However, RA muscle was different from the other muscles for the following proteins: ENO3, ALDH1A1, TPI1, PGK1, ALDOA, CRYAB, Hsp27, MLC-1F, TNNT1 and TTN. These data highlight several similarities in protein abundances of LT and TB, and of ST and SM, respectively.

The proteins with great differences among the five muscles were with the following range of variation:

MyHC-IIX: ST > SM = TB > LT > RA

ENO3: ST > SM > LT = TB > RA

TNNT1: RA > SM > TB = LT > ST

HSP27 was not different between LT, TB, ST, but it was highly abundant in RA and less abundant in SM. HSP70-1A1 was less abundant in TB and ST, but more abundant in RA and SM and intermediate in LT muscle.

LT muscle showed also some specificities since proteins like α-Tubulin were less abundant in this muscle, and not different between the four other muscles. PRDX6 was less abundant in LT and RA, and not different in the three other muscles.

### Similarities among the five muscles *via* a correlation network analysis

**Figure 3:**
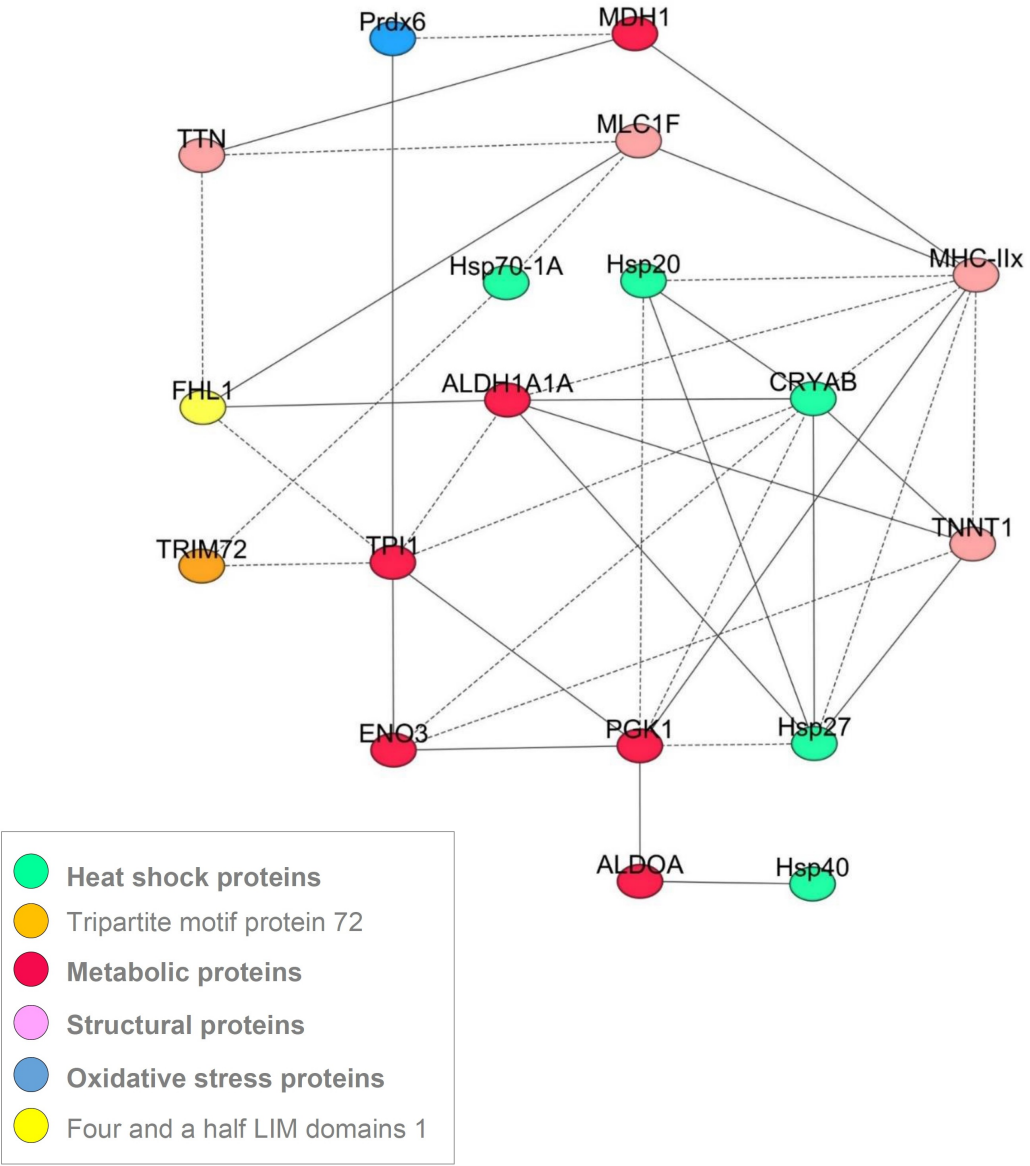
A correlation network showing the most robust correlations between the proteins obtained for the 5 muscles. The Pearson’s correlation coefficients (*P* < 0.05) between the 20 quantified proteins by RPPA on *Z*-scores were computed using the Proc CORR of SAS. The solid and dash lines represent the positive and negative correlations, respectively.

Despite the differences in the protein abundances between the five muscles, the correlation analysis using the 20 proteins allowed constructing a common correlation network based on correlations considered as robust since they were observed in all the muscles (Fig. 3). Following the procedure used by (Gagaoua et al., 2015b), a correlation between biomarkers (strength and sign) was considered robust if it existed at the same time and in the same direction for each of the five muscles. Networks describing cellular processes of the conversion of muscle into meat are very scarce (Gagaoua et al., 2015b; Picard et al., 2016). Thus, the interpretation of the consistent correlations (Fig. 3) found in this study might improve our understanding of the underlying biological pathways and interactions. The proteins the most connected with the others were in the following order MyHC-IIX (eight interactions), CRYAB (8 interactions), TPI1 and PGK1 (seven interactions), ALDH1A1 and HSP27 (six interactions), TNNT1 (five interactions), and ENO3, HSP20, FHL1, MLC1F (all with four interactions). The others showed less than four interactions and some of them were not related with any of the proteins, namely PYGB and α-Tubulin.

## Discussion

This study is, to our knowledge, the first that analyze the relative abundance of 20 proteins in five different bovine muscles sampled in dozens of animals using RPPA technique. Among these muscles and according to the large literature, SM and ST muscles are characterized as fast glycolytic, with low IMF amount and high collagen content (Jurie et al., 2007). LT, TB and RA are described as mixed fast oxido-glycolytic muscles (Gagaoua et al., 2017a; Oury et al., 2007; Picard et al., 2017a), with high IMF, and low collagen content for RA and LT (Picard et al., 2017a). This study reveals for the first time molecular similarities and specificities among the five muscles. Finally, the correlation network built using the 20 proteins highlighted robust relationships among proteins involved in beef tenderness and IMF content.

### *Rectus abdominis*, a muscle with molecular peculiarities

Very few studies on RA cattle muscle are available in the literature. The study by (Oury et al., 2010) in Charolais cattle (both steers and heifers) revealed some specificities of this muscle in comparison to TB or LT muscles. Similarly, a further recent study by our group confirmed these investigations in comparison to LT muscle of cull cows (Gagaoua et al., 2017a). These previous findings highlighted that RA muscle is characterized by higher mean cross section area of fibers than the two other muscles. In RA muscle, the slow-oxidative fibers are the largest and the fast-glycolytic the smallest (Oury et al., 2010), the inverse being generally observed (Lefaucheur, 2010; Schiaffino & Reggiani, 2011). RA muscle is also characterized by a higher proportion of slow fibers than TB and LT muscles, balanced by a lower proportion either of fast glycolytic IIX fibers (in comparison to TB muscle) or of fast oxido-glycolytic IIA fibers (in comparison to LT muscle). In accordance with these previous data, the present study confirms that the proteins of fast glycolytic type were significantly less abundant in RA muscle. On the contrary, proteins of the slow contractile type (TNNT1, ALDH1A1), and small HSP (Hsp20, Hsp27, CRYAB, Hsp70-1A) known to be more abundant in slow fibers (Gagaoua et al., 2017a; Liu & Steinacker, 2001), were the most abundant in RA in coherence with the high proportions of slow fibers, and their high cross sectional area (Gagaoua et al., 2017a; Oury et al., 2010). Overall, the results of the present study confirm the peculiarities of this muscle. Moreover, the coherent molecular peculiarities of RA when compared to conventional characteristics analysis of muscle argue for an accurate quantification of the proteins by the RPPA technique.

### Differences in protein abundances according to the contractile and metabolic properties of the muscles

The results of the present study showed high similarities in protein abundances between LT and TB and between ST and SM muscles. These two last muscles are known as fast glycolytic muscles (Totland & Kryvi, 1991). However, LT and TB were characterized as oxido-glycolytic muscles since they contain higher proportion of slow fibers, and lower proportion of fast fibers than ST and SM muscles (Picard et al., 2007). This is coherent with the higher abundance of proteins of glycolysis (ENO3, PGK1 and ALDOA) and fast contractile type (MyHC- IIX) and to the lower abundance of slow type protein such as TNNT1 in ST and SM, comparatively to LT and TB. These results are in accordance with the comparison between LT and ST muscle in Charolais males (Guillemin et al., 2011b) or in different continental young bulls (Gagaoua et al., 2015b). The results demonstrate also some specificities in the abundance of small HSP proteins, which were more abundant in the muscles with higher proportions of slow fibers (RA and LT) in comparison to ST, SM and TB muscles. This corroborates the results of Guillemin et al. (2011a) and Guillemin et al. (2011b) showing a higher abundance of small HSP in LT than in ST muscles, in line with the high abundance of these proteins in slow fibers (Liu & Steinacker, 2001). These findings are further in agreement with those obtained on young bulls (Gagaoua et al., 2015b).

According to the results of the present study, PRDX6, an anti-oxidant and cell-protective protein, is less abundant in the most slow oxidative muscles LT, RA, and more abundant in the three other muscles, without any differences among them. Similar results with a lower abundance in LT than ST muscle were also observed in our laboratory (Guillemin et al., 2011b). Accordingly, the positive correlations observed between PRDX6 and ENO3 or TPI1 involved in glycolysis, and a negative correlation with MDH1 involved in oxidative pathway, is coherent with the low abundance of PRDX6. In agreement with a previously proposed muscle-related regulation (Gagaoua et al., 2015a; Knoops et al., 1999), the study of (Keady et al., 2013) comparing *longissimus lumborum* from Aberdeen Angus (AA) characterized by slow oxidative muscles, and Belgian Blue (BB) characterized by fast glycolytic muscles, observed higher abundance of metabolic enzymes involved in glycolysis and the citric acid cycle in AA *vs.* BB steers. Consequently, PRDX6 showed increased abundance in muscle of AA relative to BB steers. PRDX6 is a bifunctional protein with both glutathione peroxidase and phospholipase A2 (PLA2) activities (Fisher, 2017) that was recently found to play a great role in meat tenderness determinism (Gagaoua et al., 2015b) and pH decline (Gagaoua et al., 2015c). In a recent study by our group, we found a positive correlation between PRDX6 and µ-calpain irrespective of muscle and breed (Gagaoua et al., 2015b), and we suggested PRDX6 to play a pivotal role in the protection of proteases, including µ-calpain, from proteolysis (Rowe et al., 2004). In fact, it has been reported that different muscles exhibited differential protein synthesis responses to oxidative stress (Salo, Donovan & Davies, 1991).

### Proteins without muscle effect

Among the 20 studied proteins, four proteins only (HSP40, FHL1, PYGB and MDH1) were similar in their relative abundances irrespective of the muscle (Table 2). In a recent study using the same breed, we have reported similar finding for HSP40 in RA and LT muscles (Gagaoua et al., 2017a). For MDH1, similar findings were reported between LT and ST muscles of young bulls (Picard et al., 2014) and steers (Guillemin et al., 2011a). As stated above, the studies that investigated differences in protein abundance according to cattle muscles are very scarce. However, it is worth noticing that none or less protein-correlations were found for PYGB (no interaction), HSP40 (one interaction), MDH1 (three interactions) and FHL1 (four interactions) compared to the other proteins (Fig. 3). Thus, further investigations are needed to understand these results and study their particular use as baselines for comparisons or quantification of protein using high throughput technique such as RPPA.

### Relationships among the proteins: the correlation network

Several recent reviews have been published relating skeletal muscle fiber type to meat quality traits (Joo et al., 2013; Lefaucheur, 2010; Picard et al., 2010; Picard et al., 2002). The present study proposed to gain insight in these relations by the means of a correlation network obtained using the 20 protein abundances within five muscles differing in fiber types and metabolism. A correlation between two proteins (strength and sign) was considered robust if it existed at the same time and in the same direction for the five muscles. The proteins the most connected with the others were MyHC-IIX (eight interactions) and CRYAB (eight interactions). Myosin heavy chains are well known to play great roles in meat tenderness determinism (Picard et al., 2010). The relationship of MyHC-IIX with several proteins confirms our recent findings (Gagaoua et al., 2015b). The findings of this work are consistent with existing results stating that the contractile and metabolic properties of muscle play a major role in the elaboration of beef qualities (Chriki et al., 2013; Gagaoua et al., 2016; Picard et al., 2014).

Among the three small HSP, CRYAB is connected with a high number of proteins comparatively to HSP27 (six correlations) and HSP20 (four correlations). Similar findings were reported in the first correlation network for cattle muscle (Gagaoua et al., 2015b). The three small HSP proteins were indirectly interrelated, through HSP27. Small HSP are expressed at low levels in muscle until an inducible event (Sugiyama et al., 2000). The higher number of correlations of CRYAB could be explained by its implication in various biological functions. It is for example known to act as a molecular chaperone preventing aggregation of partially folded polypeptides, in the negative regulation of intracellular transport and apoptotic process, protein homodimerization activity, cellular differentiation and proliferation, translation, oxidative stress regulation, and cytoskeleton stabilization (Kamradt, Chen & Cryns, 2001; Malin et al., 2014). CRYAB is necessary for the maintenance of myofiber size, as mice KO for CRYAB exhibit satellite cell hyperplasia and myofiber hypotrophy (Neppl, Kataoka & Wang, 2014). Moreover, CRYAB was associated with all three cytoskeletal networks: microfilaments, microtubules and intermediate filaments and specifically with desmin (reviewed in Wettstein et al., 2012) and was proposed to play great role in the tenderizing process of meat (Lomiwes et al., 2014a; Picard & Gagaoua, 2017). Furthermore, desmin and CRYAB are localized at sarcoplasmic reticulum–mitochondria-associated membranes where they interact with the core component of mitochondria suggesting that these associations could be crucial in mitoprotection (Diokmetzidou et al., 2016). Accordingly, (Lomiwes et al., 2014b) found, using *in vitro* studies, that CRYAB interacted with desmin, titin, HSP20, HSP27 and µ-calpain in bovine muscle. All these data demonstrate that CRYAB plays an important role in skeletal muscle homeostasis. The negative correlations of CRYAB with fast or glycolytic proteins, and the positive correlation with a slow contractile protein, are coherent with the data of the literature. They further corroborate the differences observed among the five studied muscles, with a higher abundance in RA muscle. The positive correlation with HSP27, is also coherent with data of the literature (Arrigo et al., 2007; Gagaoua et al., 2015b; Lomiwes et al., 2014a).

HSP27-encoded by *HspB1* is constitutively present in a wide variety of tissues and in many cell lines. The abundance of HSP27 is greater in skeletal muscle, indicating an important role for muscle physiology (Kammoun et al., 2016) and thus for muscle to meat conversion (Picard & Gagaoua, 2017; Picard, Gagaoua & Hollung, 2017b). An earlier study by our group showed that it is at a crucial hub in a functional network involved in beef tenderness (Guillemin et al., 2011a). In *HspB1*-null mice, comparative proteomics in *Tibialis anterior* muscle comparatively to the littermate controls showed that the proteins impacted by the absence of HSP27 belong mainly to calcium homeostasis (DRL and CALSQ1), contraction (TnnT3), energy metabolism (TPI1, MDH1, PDHB, CKM, PYGM and APOA1) and Hsp proteins family (HspA9) (Picard et al., 2016). The negative correlation observed in the present study between HSP27 and slow oxidative proteins such as TNNT1, is in accordance with the data of these authors (Table S1).

TPI1 is a glycolytic enzyme playing an important role in energy generation for muscle cells; it catalyzes the interconversion of dihydroxy-acetone phosphate and glyceraldehyde 3-phosphate, substrate directly involved in the glycolytic pathway. Gene Ontology annotations related to this gene include ubiquitin protein ligase binding and triose-phosphate isomerase activity. TPI enzyme is necessary for cell growth and maintenance (Chen et al., 2017). The TPI1 gene was significantly up-regulated in high marbling cattle (Shin & Chung, 2016). It was further proposed as a potential biomarker for IMF (Kim et al., 2008), color stability and development during storage in beef (Gagaoua et al., 2017a; Gagaoua et al., 2017b) and ultimate pH (Gagaoua et al., 2017a). Its positive correlation with ENO3 and PRDX6 may involve cellular stress response under hypoxia or low glucose levels (Sedoris, Thomas & Miller, 2010). In the present study, TPI1 was negatively correlated with FHL1 (Fig. 1 and Table S1). It was reported that FHL plays a role in fat deposition (Wang et al., 2009), which may explain its link with TPI. *FHL1* gene consists of eight exons, giving rise to three protein isoforms: FHL1A, FHL1B, and FHL1C (Gueneau et al., 2009). FHL1A is the predominant isoform in skeletal and cardiac muscle, and comprises an N-terminal half LIM domain followed by four complete LIM domains. FHL1 is a member of the four-and-a-half-LIM-only protein family (Kadrmas & Beckerle, 2004). FHL1 regulates gene transcription, cell proliferation, metabolism and apoptosis (Shathasivam, Kislinger & Gramolini, 2010). The LIM domain forms a tandem zinc-finger structure that provides a modular protein-binding interface, through which FHL1 functions as adaptor or scaffold to support the assembly of multimeric protein complexes and regulate the localization and activity of their partners (Shathasivam, Kislinger & Gramolini, 2010). This protein is confined to the Z-line of skeletal muscle and its proteolysis is linked to the release of intact α-actinin from bovine myofibrils and contributes to the weakening of the Z-line during meat tenderizing (Morzel et al., 2004). FHL1 may also interact with other biological pathways, namely metabolic enzymes (Gagaoua et al., 2018a; Raskin et al., 2012), in accordance with the correlations observed in the present study. FHL1 expression is high in striated muscles and has been suggested to play an important role in skeletal muscle growth and remodeling (Cowling et al., 2008). Moreover, this protein was recently revealed as a novel regulator of calcium homeostasis (Pillar et al., 2017).

In the present study, TPI1 was also positively correlated with PGK1, another glycolytic enzyme, which was positively correlated with three glycolytic enzymes and negatively with the three sHSP proteins. PGK1 is the first ATP-generating enzyme in the glycolytic pathway, catalyzing the conversion of 1,3-diphosphoglycerate to 3-phosphoglycerate. This enzyme is upregulated in many human cancers and has recently been shown to translocate to the mitochondria, where it specifically phosphorylates pyruvate dehydrogenase kinase (Li, Zheng & Lu, 2016). The encoded protein has been identified as a moonlighting protein based on its ability to perform mechanistically distinct functions. Deficiency of the enzyme is associated with a wide range of clinical phenotypes. In addition to its role as a glycolytic enzyme, it seems that PGK1 acts as a polymerase α-cofactor protein (primer recognition protein). In this study, it was correlated with the three studied small HSP proteins. This relation may be explained by the presence of a heat shock element in the core of this protein (Piper et al., 1988). In pigs, PGK1 was reported to be increased in abundance following acute heat stress (Cruzen et al., 2015). These would be attributed to *p-m* cell status, namely the link with nutrients decline (glucose) after animal bleeding and hypoxia, and thus to apoptosis set off (Ouali et al., 2013). HSP40 was reported to link with another enzyme kinase, pyruvate kinase M2, and to regulate glycolysis (Huang et al., 2014). It is known that HSPs are up-regulated while cells are exposed to glucose supply or oxygen deprivation (Sterrenberg, Blatch & Edkins, 2011). This leads us to suggest that during the first hours of muscle *p-m*, sophisticated mechanisms may occur, such as the regulation of glucose metabolism *via* different protein pathways associations, for instance HSP-metabolic enzymes (PGK1). Those involvements in cattle muscle, namely with pH decline, are in accordance with the recent data of our group (Gagaoua et al., 2018a; Gagaoua et al., 2015c).

## Conclusion

These results are the first to analyze simultaneously 20 proteins in five different bovine muscles by using a high-throughput immunological technique that is not yet widely applied in the field of muscle biology. The results produce new insights, using a local breed (Rouge des Prés) about the relationships between proteins biomarkers of tenderness and intramuscular fat content in several muscles differing by their contractile and metabolic properties, and characterized by different sensory qualities. This new knowledge is important for understanding the mechanisms related to tenderness or fat deposition according to the muscle, two meat properties of importance for consumer acceptability and thus for the economy of the beef industry. However, the data need further confirmation using other cattle breeds and animal types following the same innovative technical tool.

##  Supplemental Information

10.7717/peerj.4891/supp-1 Table S1 Robust Pearson correlation coefficients obtained for the five muscles and for each muscle alone that were used to build the correlation network of Fig. 3 The red and green rows highlight the significant negative or positive correlations, respectively.

10.7717/peerj.4891/supp-2 Data S1 Raw protein data Raw protein data of the 20 muscle proteins quantified by Reverse Phase Protein Array (RPPA) in *Longissimus thoracis* (LT), *Semimembranosus* (SM), *Rectus abdominis* (RA), *Triceps brachii* (TB) and *Semitendinosus* (ST) and expressed in log2 transformed values.

## Additional Information and Declarations

### Data Availability

The following information was supplied regarding data availability: The raw data are provided in the Supplemental Files.
